# Hematology and Plasma Biochemistry Reference Values of the Subgenus *Hapturosaurus* (*Varanus macraei*, *Varanus prasinus*, *Varanus beccarii*) Under Human Care

**DOI:** 10.3390/vetsci12050454

**Published:** 2025-05-09

**Authors:** Jorge Sobrino-Yacobi, Manuel Fuertes-Recuero, Manuel de la Riva-Fraga, Teresa Encinas Cerezo, Andrés Montesinos Barceló, Álvaro Camina Vega, Pablo Morón-Elorza

**Affiliations:** 1 Complutense Veterinary Teaching Hospital, Complutense University of Madrid, Avda. Puerta de Hierro s/n, 28040 Madrid, Spain; jorsobri@ucm.es (J.S.-Y.); tencinas@vet.ucm.es (T.E.C.); 2 Department of Physiology of the Faculty of Veterinary Medicine, Complutense University of Madrid, Avda. Puerta de Hierro s/n, 28040 Madrid, Spain; madelariva@faunia.es; 3 Faunia, Avenida de las Comunidades 28, 28032 Madrid, Spain; 4 Department of Pharmacology and Toxicology of the Faculty of Veterinary Medicine, Complutense University of Madrid, Avda. Puerta de Hierro s/n, 28040 Madrid, Spain; pmoron01@ucm.es; 5 Exotic Animals Veterinary Hospital Medivet-Los Sauces, Santa Engracia 63, 28010 Madrid, Spain; amontesinosbarcelo@gmail.com; 6 Atrox Group, Faunia, Avenida de las Comunidades 28, 28032 Madrid, Spain; a.camina@grupoatrox.com; 7 International Union for Conservation of Nature Species Survival Commission Monitor Lizard Specialist Group, 28 Rue Mauverney, 1196 Gland, Switzerland; 8 Fundación Oceanografic de la Comunitat Valenciana, Carrer d’Eduardo Primo Yúfera, 1, 46013 Valencia, Spain

**Keywords:** reptiles, tree monitors lizards, blood analysis, reference intervals

## Abstract

The tree monitor lizards of the subgenus *Hapturosaurus* are an understudied but increasingly important group of reptiles in zoos. The routine blood analysis of reptiles in captivity is highly recommended; however, the lack of reliable reference values for many species severely limits their clinical use. This study aims to establish preliminary reference values (RV) for the main hematological and biochemical parameters in three species of the subgenus *Hapturosaurus* (*Varanus macraei*, *Varanus prasinus* and *Varanus beccarii*). Blood samples were taken from healthy adult individuals (*n* = 34), females (*n* = 18) and males (*n* = 16), maintained under controlled conditions in a zoo. A complete biochemical analysis was performed on all individuals, including alanine-aminotransferase, alkaline phosphatase, gamma-glutamyl transpeptidase, aspartate-aminotransferase, creatine kinase, bile acids, urea, uric acid, total solids, total proteins, albumin, glucose, fructosamine, lactate, cholesterol, triglycerides, amylase, calcium, phosphorus, calcium:phosphorus ratio, and magnesium. In addition, a complete hematological analysis was conducted on these individuals, including hematocrit, hemoglobin concentration, total erythrocyte and leukocyte counts, and leukocyte differential count. From the data obtained, reference intervals were established following the guidelines provided by the American Society for Veterinary Clinical Pathology (ASVCP). In this study, the results of the three species were unified to increase the sample size and to obtain more representative reference intervals. This unification was supported by a statistical analysis using the one-factor ANOVA test, which showed no statistically significant differences between species (*p* < 0.050). These data constitute an essential first step for the clinical practice and management of these *Hapturosaurus* species in zoos and specialized centers. Future collaboration with other institutions will help expand the dataset and enhance the accuracy of the established reference values.

## Simple Summary

Tree monitor lizards of the *Hapturosaurus* group are becoming increasingly common in zoos, but there is little scientific information to guide veterinarians in assessing their health. This study aimed to establish initial blood reference intervals in three species of these lizards: the green tree monitor, the black tree monitor and the blue tree monitor. Blood samples from healthy adult animals kept in controlled conditions were carefully analyzed. The results included measurements of hematology and biochemistry parameters. No significant differences were found between the three species, allowing the results to be combined for stronger conclusions. By having reference values for key health parameters following the guidelines provided by the American Society for Veterinary Clinical Pathology (ASVCP), veterinary professionals can identify health issues at an earlier stage, thereby improving the overall care and management of these animals. Additionally, this research supports conservation efforts by aiding zoos and captive breeding centers in maintaining healthy populations of tree monitor lizard species, many of which are threatened in their natural habitats.

## 1. Introduction

Reference values (RV) for hematology and plasma biochemistry are essential for the interpretation of clinical laboratory test results in any species, and even more so in wildlife medicine, as they allow an objective assessment of the health status of individuals; they are expressed as reference intervals (RI) covering 90–95% of a healthy reference population [1,2]. The optimal method for determining RV recommends a species-specific population of 120 individuals; however, when access to a large and homogeneous population of healthy individuals under similar environmental conditions is limited, a smaller number of reference individuals may be used. Although the uncertainty in reference interval (RI) estimation decreases as sample size increases, a sample of 20–40 reference individuals is sufficient to establish RIs using parametric or robust statistical methods [1,2,3].

Reptile medicine has developed significantly in recent decades due to their increasing popularity as pets and the interest of zoos and conservation centers. Most of their diseases are the result of inadequate handling and husbandry conditions, so it is necessary to provide them with appropriate environmental characteristics [4]. Blood sampling should be included in the routine health assessment of reptiles maintained under human care [5,6,7,8]. These tests can detect changes in organ function, metabolic imbalances and immunological responses, making them essential for the diagnosis, monitoring and prognosis of diseases. The hemogram is an essential tool for detecting pathologies such as anemia, inflammatory diseases, parasitosis, hematopoietic disorders and hemostatic alterations [9]. In addition, the continuous monitoring of hematological parameters can provide valuable information on treatment efficacy and disease progression [10]. In reptile clinical diagnostics, certain biochemical parameters such as total protein, glucose, uric acid, aspartate aminotransferase (AST), calcium and phosphorus are routinely assessed due to their relevance in evaluating organ function, nutritional status, and overall health [11]. A comprehensive biochemical profile, encompassing these and additional parameters, allows for a thorough assessment of renal and hepatic function, glucose, protein and lipid metabolism, reproductive function and electrolyte balance [9,11]. Therefore, the determination of RV is important for veterinarians, as it allows for the clinical interpretation of analytical results by comparing patient data with established ranges for healthy individuals, being an essential tool for guiding diagnostic and treatment decisions [3,12]. Reference ranges for reptiles are still very sparse compared to other animals [5,6,7]. Furthermore, in these species, obtaining and using reference ranges poses significant challenges and limitations due to the large variability in results, which can be attributed to a combination of intrinsic and extrinsic factors that can influence baseline values [13]. Intrinsic factors include species, sex, age and physiological condition, while extrinsic factors include diet, ambient temperature, season, habitat and stress [14]. Published reference ranges for reptiles often lack essential information, especially regarding the environmental conditions of the study population [9]. Therefore, reference values must be specific and consider the characteristics of animals kept under particular environmental conditions, as these values can vary even within the same species [1,2]. Despite these limitations, reference values remain indispensable, and their establishment and interpretation must be carried out by controlling the variables that affect the measurement of the parameters studied and complemented by the clinical judgement of the veterinarian [1,3].

The family Varanidae contains a single genus, *Varanus* (divided into eleven subgenera), with 86 species of lizards known as monitor lizards (*Varanus* spp.). Monitor lizards are part of a clade called Toxicofera, which also includes a large number of lizard species and snakes [15]. Within the genus *Varanus*, one of the subgenera that stands out is the subgenus *Hapturosaurus*, which may be translated as ‘prehensile-tailed lizard’. It contains nine species of medium-sized monitor lizards found in the tropical rainforests of Indonesia and Papua New Guinea, characterized by a variety of adaptations to arboreal life [16,17,18]. Within this subgenus, three species are of particular interest due to their increasing presence in zoological centers: *Varanus prasinus*, known as the green tree monitor; *Varanus beccarii*, or black tree monitor; and *Varanus macraei*, the blue tree monitor, the largest species in the subgenus [16]. There is a close genetic relationship between these species. In fact, throughout the 20th century, *Varanus prasinus* was a polytypic complex comprising several subspecies, including *Varanus beccarii* [19]. According to the International Union for Conservation of Nature (IUCN) Red List [20], the species in the subgenus are threatened to varying degrees by restricted distribution, deforestation and commercial exploitation; *Varanus macraei* is listed as Endangered, *Varanus prasinus* as Least Concern and *Varanus beccarii* as Data Deficient. High mortality during capture and export exacerbates the vulnerability of these species, highlighting the urgent need for conservation strategies that include habitat protection, trade regulation and captive breeding, supported by scientific research to drive effective actions [21,22,23]. Research on these species remains limited, with most studies primarily focusing on breeding in zoological institutions [24,25,26] or microbiological studies investigating bacterial and viral pathologies in these species [27,28,29].

Hematological and blood biochemical RV have been described in some reptile species, including those within the *Varanus* genus, such as the Nile monitor (*Varanus niloticus*), Asian water monitor (*Varanus salvator*), savannah monitor (*Varanus exanthematicus*), Dumeril’s monitor (*Varanus dumerilii*), lace monitor (*Varanus varius*) and Komodo dragon (*Varanus komodoensis*) [5,6,7,8,30,31,32,33]. Furthermore, due to the challenges of accessing a large and homogeneous *Varanus* population, most studies describing hematological and plasma biochemical RV in these animals are often based on small study populations (often less than 20 individuals) [5,6,7]. However, to the authors’ knowledge, RVs have not yet been determined for any species of the subgenus *Hapturosaurus*. This lack of data severely limits the diagnostic tools available to veterinarians and professionals working with these species, highlighting the need for species-specific research to establish reliable and applicable reference values.

The aim of this study was to establish RVs for key hematological and biochemical values in three species of the subgenus *Hapturosaurus* (*Varanus macraei*, *Varanus prasinus*, *Varanus beccarii*) kept under human care, and to evaluate any gender-related differences. Additionally, the study aims to provide a preliminary guide for veterinarians in the treatment and clinical monitoring of these species, and to compare these values with those obtained for other *Varanus* species. This information will enable the more accurate interpretation of blood analysis results and enhance the quality of health assessments for these species.

## 2. Materials and Methods

This study used a population of *Hapturosaurus* species (*Varanus macraei*, *Varanus prasinus*, *Varanus beccarii*) maintained in the facilities of Faunia (Madrid, Spain; https://www.faunia.es/; accessed on 30 January 2025). All procedures included in this study were part of the regular veterinary check-ups performed by the zoo’s veterinary team on the animals in their care. No additional clinical procedures were required for data collection in this study. All procedures were conducted in compliance with animal welfare regulations (European Parliament and Council Directive 2010/63/UE, 22 September 2010). Data from the monitor lizard species were collected from March 2023 to April 2024 avoiding the reproductive season.

An initial group of 34 individuals of three *Varanus* species of the subgenus *Hapturosaurus* were included in this study, of which 17 individuals (6 males and 11 females) were *Varanus macraei*, 13 individuals (8 males and 5 females) were *Varanus prasinus,* and 4 individuals (2 males and 2 females) were *Varanus beccarii*. Individuals included in the study were uniquely identified using microchips, with the corresponding information recorded for sample organization. These data are described in Appendix A.1.

All animals underwent a detailed veterinary examination and a thorough review of their medical history. Individuals of these three species that met the following criteria were included in the study: adult animals (defined as those over 2 years old) with a minimum weight of 110 g and a minimum total length of 60 cm, with no previously diagnosed pathologies and no ongoing medical treatment. Furthermore, animals were excluded from the study if, during the physical examination prior to blood sampling, they showed clinical signs compatible with any pathology.

All animals were maintained at Faunia and were housed individually or in pairs in terrariums of at least 120 cm × 90 cm × 50 cm (length × height × depth). The terrariums were provided with a substrate of coconut fiber and dry leaves, which was sprayed daily with fresh water. Feces and other debris were removed daily to keep the substrate clean. Terrarium dimensions were recorded, as well as environmental conditions, including diurnal temperature range (in degrees Celsius) and relative humidity. A laser thermo-hygrometer (UNI-T UT333; Dongguan, China) was used for these measurements. The values obtained are described in Appendix A.2. The diet consisted of live food, mainly Argentine cockroaches (*Blaptica dubia)*, supplemented once a week with superworms (*Zophobas morio* larvae) and larvae of sun beetles (*Pachnoda butana*). These individuals were fed three times a week and received a supplement at each feeding, alternating between calcium carbonate, vitamin and mineral supplement (Nekton^®^-Rep; Keltern, Germany), and calcium, phosphorus and vitamin D3 supplement (Nekton^®^-MSA; Keltern, Germany).

Initially, a general physical examination was performed, and weight and length data were obtained. The physical examination assessed parameters such as body condition, skin condition, presence of external lesions, mobility and posture, and response to external stimuli. In addition, the degree of hydration was assessed by observing the turgor and elasticity of the skin, as well as the thickness of the saliva and the absence of sinking of the eyeball. The body weight (g) was determined using a crane scale (range 5 kg/precision 0.01–1 g; Covetrus^®^; Portland, ME, USA) and length (cm) was measured for total length (snout to tip of tail; TL) and snout to vent (SVL) using a flexible nylon measure. These data are given in Appendix A.1.

**Figure 1:**
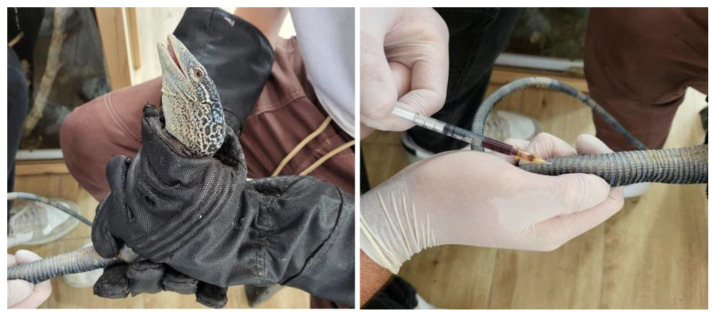
Animal handling and blood collection in a blue tree monitor lizard (*Varanus macraei*).

Blood samples were obtained by the zoo veterinarians as part of the veterinary checks-ups carried out on these species at the zoo. All individuals were fasted for 24 h before sampling. For veterinary handling, it was necessary to capture and restrain the animals using protective gloves specifically designed for handling reptiles. Two veterinarians were required to perform the blood collection: one to restrain the animal and the other to collect the blood (Figure 1). Previous studies have reported that 0.4–0.8 mL of blood per 100 g of body weight can be safely collected from healthy Varanus [13]. Given that the mean weight of the study individuals was 244 g, 1 mL of blood was collected by caudal venipuncture at the base of the tail via a ventral midline approach using a 25-gauge needle attached to a 1 mL heparinized syringe (Fibrilin 20 IU/mL; Laboratorios Rovi; Madrid, Spain) after disinfection of the area with 96° sanitary alcohol.

Samples were immediately transferred into 1 mL lithium heparin tubes (TP010010, Everest-tecnovet; Barcelona, Spain), which were refrigerated at 4 °C and processed within a maximum of 120 min after collection. The time from capture to blood collection was always less than 5 min (range 2 to 5 min). None of the animals showed any clinical signs either during the study or in the month following the study.

The manual packed cell volume (PCV) was determined using microhematocrit tubes, centrifuged at 1400× *g* (3500 rpm) for 6 min at room temperature (24 °C) in a centrifuge (Microcen^®^ 24, Ortoalresa-Álvarez Redondo; Daganzo de Arriba, Spain). The plasma total solids (TS) were determined with an analog refractometer (Comecta^®^ model C-6; Comecta; Barcelon, Spain). Hemoglobin was determined using a portable hemoglobinometer (HemoCue^®^ 201 system; HemoCue AB; Ängelholm, Sweden). Erythrocyte indices including mean corpuscular volume (MCV), mean corpuscular hemoglobin (MCH), and mean corpuscular hemoglobin concentration (MCHC) were employed following previous studies [5,8,10].

For white blood cell (WBC) and red blood cell (RBC) counts, samples collected in 2023 and 2024 were processed differently. As immediate cell count was not possible in 2023, for the 2023 samples, a 50 μL aliquot of the blood was diluted (1:5 dilution) by adding 200 μL of 10% neutral buffered formalin to preserve the blood cells and allow cell counts to be made over a longer period [34,35]. The formalin-fixed blood aliquots were stored at ambient temperature and processed within the next 30 days after collection. During processing, 20 µL of formalin-fixed cells (1:5) was combined with 380 µL of Natt–Herrick’s solution (NH) (1:20), achieving a final dilution of 1:100. After 15 minutes of mixing, RBC and WBC counts were conducted [36].

Because of the poor results obtained via the formalin preservation protocol for the studied species, the samples collected in the 2024 sampling season were directly processed after collection by mixing 5 μL of fresh whole blood with 995 μL of NH within 30 min of collection, achieving a final dilution of 1:200. White and red blood cell counts were performed using the same methodology as was used in 2023 [36].

Red blood cells (RBCs) and total granulocytes were manually counted using a Neubauer Improved counting chamber (Neubauer-Improved, Laboroptik; Lancing, UK). A 5 µL blood sample was mixed with 995 µL of Natt–Herrick’s solution (Ref 004025-0500, Bioanalytic GmbH; Umkirch, Germany). The chamber was examined under a microscope using a 40× lens (Leica^®^ DME, Leica Microsistemas; Madrid, Spain), and cells were counted within the designated grids. The total count was then adjusted using the dilution factor and chamber volume to determine the final cell concentration.

In addition, blood smears for all 2023 and 2024 samples were prepared using the lithium–heparin fresh blood samples, previously homogenized, within 120 min of collection. After preparation, the slides were dried at ambient temperature (24 °C) and stained by two different methods: Diff-Quik staining (Rapid Panoptic Kit 3 × 250 mL, MAIM; Barcelona, Spain) and May Grünwald-Giemsa staining (May Grünwald and Giemsa, Química Clínica Aplicada; Amposta, Tarragona). Leukocyte differentiation was also performed on the blood smears using a microscope equipped with a 100× lens and immersion oil. The procedure involved observing the stained smears, identifying and counting at least 100 leukocytes per animal and sample, and classifying them according to their morphology, cytoplasmic and nuclear characteristics as lymphocytes, heterophils, monocytes, eosinophils and basophils. The relative percentages of each cell type in the sample were calculated.

Once the hematological analyses were completed, the heparin tubes containing the remaining blood were centrifuged within 120 min of collection at 448 g for 5 min. The plasma was separated, collected in Eppendorf tubes, and stored at −20 °C until processing. All samples were processed within a maximum of 30 days from collection at the laboratory located at the veterinary clinic in Faunia, using a multi-functional benchtop clinical chemistry analyzer (Mindray BS-230, Mindray; Shenzhen, China) equipped with biochemistry commercial kits (Clonatest^®^, Linear Chemicals; Barcelona, Spain). The plasma biochemistry profiles included glucose (mg/dL), uric acid (mg/dL), urea (mg/dL), total cholesterol (mg/dL), triglycerides (mg/dL), calcium (mg/dL), phosphorus (mg/dL), magnesium (mg/dL), aspartate aminotransferase (AST; U/L), creatine kinase (CK; U/L), amylase (U/L), alkaline phosphatase (U/L), alanine aminotransferase (ALT; U/L), gamma-glutamyltransferase (GGT; U/L), bile acids (μmol/L), total protein (g/dL), albumin (g/dL), fructosamine (μmol/L) and lactate (mg/dL).

Reference intervals were calculated according to the recommended guidelines of the American Society of Veterinary Clinical Pathology (ASVCP) using the Reference Value Advisor 2.0 excel add-in and MedCalc^®^ statistical software (version 23.2.1) [1,12]. Outliers were identified using Dixon’s outlier range statistic, and histograms were analyzed [37]. If an outlier was detected for a single parameter, it was excluded from the analysis; if three or more parameters in the same sample showed outliers, the data for that individual were discarded.

The basic statistical analysis of quantitative variables included the calculation of arithmetic mean, standard deviation (SD), median, maximum and minimum values. In addition, before continuing with the statistical analysis, all variables were tested for normality and homoscedasticity using the Anderson–Darling test and Levene’s tests, respectively, along with visual assessments such as via histograms. Based on the results obtained, to evaluate the influence of species (*V. macraei*, *V. prasinus* and *V. beccarii*), an analysis of variance (ANOVA) and a Kruskal–Wallis test were performed for those variables with a normal distribution (HTC, Hb, RBC, MCV, HCM, CHCM, WBC, heterophils, lymphocytes, monocytes, bile ac-ids, urea, total solids, total proteins, albumin, glucose, fructosamine, lactate, cholesterol, amylase, calcium, phosphorus and magnesium) and for variables with non-normally distributed data (Basophils, CK, triglycerides and uric acid), respectively. A standard parametric method was used for all parameters to calculate RI, except creatine kinase, uric acid and triglycerides, for which a robust parametric method was used instead. Where ANOVA revealed statistically significant differences, Tukey’s post hoc test was used for comparisons between pairs of species. As no significant differences between species were found, all subsequent data analysis was performed by considering a pool of the values of all individuals (regardless of species), thus increasing the sample size and establishing more representative reference intervals. For comparison between sexes, a Student’s test and a Mann–Whitney U-test were used for variables with or without normal distribution, respectively. All statistical analyses were performed using IBM SPSS Statistics 28.0 software. The level of significance was set at *p* < 0.05.

## 3. Results

### 3.1. Morphometric Measurements

The mean weight of the animals was 243.5 g (range: 111.0–530.0 g), with a mean total length (TL) of 78.8 cm (range: 64.0–106.0 cm) and a mean snout to vent length (SVL) of 27.9 cm (range: 23.0–35.0 cm). The individual results of the morphometric parameters are described in Appendix A.1. Among the species analyzed, *Varanus macraei* showed the highest values with a mean weight of 279.3 g (range: 148–530 g), a mean TL of 84.2 cm (range: 65.0–94.0 cm) and a mean SVL of 29.6 cm (range: 27.0–35.0 cm), followed by *Varanus beccarii* with a mean weight of 261.3 g (range: 223.0–298.0 g), a mean TL of 81.3 cm (range: 78.5–86.0 cm) and a mean SVL of 28.3 cm (range: 27.0–29.5 cm), followed by *Varanus prasinus* with a mean weight of 191.2 g (range: 11.0–305.0 g), a mean TL of 71.0 cm (range: 61–79 cm) and a mean SVL of 25.6 cm (range: 23–27 cm). Significant differences were found between *Varanus macraei* and *Varanus prasinus* for weight (*p* = 0.013), TL (*p* < 0.010) and SVL (*p* < 0.010) using a one-factor ANOVA test and a Tukey post-hoc test. Females had a mean weight of 216.7 g, a mean TL of 77.9 cm and a mean SVL of 27.8 cm, while males had higher values of 273.6 g, 79.8 cm and 28 cm, respectively. However, no statistically significant differences were found between the sexes for weight, TL, and SVL (*p* = 0.055, *p* = 0.575 and *p* = 0.858, respectively; Student’s *t*-test).

No statistically significant differences were detected for any of the hematological and plasma biochemistry values obtained from the different species of the subgenus *Hapturosaurus* (*Varanus macraei*, *Varanus prasinus*, *Varanus beccarii*) (*p* > 0.050; ANOVA of one factor; Appendix A.3).

### 3.2. Hematology

Descriptive statistics for hematology are presented in Table 1. The parameters hemoglobin, erythrocyte count, and erythrocyte indices including mean corpuscular volume (MCV), mean corpuscular hemoglobin (MCH), and mean corpuscular hemoglobin concentration (MCHC), as well as leukocyte count, have sample sizes of fewer than 20 individuals due to the imprecision of results derived from samples preserved in formalin. Blood preservation with formalin appeared to degrade the samples from all three *Varanus* species studied, and as a result, all counts performed using this method were discarded from the hematological analysis. Therefore, the ranges presented in Table 1 for these parameters, marked with an asterisk, should be interpreted with caution due to the smaller sample size. As indicated by the ASVCP guidelines for parameters with sample sizes under 20 animals, individual results for these hematological parameters are provided in Appendix A.4.1.

A formalin preservation protocol described by Arnold et al. (2014) was employed during the first sampling season in 2023, as this technique had been successfully used in other species when direct hematology analysis was not feasible. It has proven effective in preserving cells and minimizing clumping, allowing samples to be analyzed days or even weeks after collection [34]. Following the poor initial hematology results obtained in 2023, and to evaluate the suitability of this preservation protocol for reptiles, serial granulocyte counts were performed on formalin-preserved blood samples (Appendix A.4.2). The results reveal a progressive decline in cell counts over time, indicating degradation and confirming that the protocol was ineffective for preserving blood cells in reptiles. As a result of the imprecision of these findings, all RBC and WBC counts obtained using this method for the samples collected in 2023 were discarded and the preservation protocol was not used in the sampling season in 2024.

In this study, the leukocyte differential of blood samples was performed using two different stained smears: Diff-Quik and May-Grünwald Giemsa. After statistical analysis, no statistically significant differences were found between the results obtained with the two methods (*p* > 0.005; Student’s *t*-test). Results for both stains are provided in Table 2.

In the leukocyte differential, the results show a higher proportion of lymphocytes than other white blood cells, with an average of 54.1% (±10.4), followed by heterophils, with an average of 38.9% (±11.6), and finally the minority cell populations consisted of monocytes and basophils, with averages of 6.9% (±4.3) and 0.1% (±0.5), respectively.

We did not find statistically significant differences between sexes for any hematological value (*p* > 0.050; Student’s *t*-test; Table 3).

### 3.3. Plasma Biochemistry

Descriptive statistics for blood chemistry are presented in Table 4.

Individual results for these clinical chemistry parameters are presented in Appendix A.5. We found no statistically significant differences between the sexes for any of the plasma biochemical values, except for cholesterol, triglycerides and bile acids, which were higher in females (*p* < 0.001, *p* = 0.016, *p* = 0.010, respectively, Table 5).

## 4. Discussion

The available studies on hematological and blood biochemical parameters in reptiles are very limited compared to other domestic and exotic species. This lack of information is characterized by drastic variations in reference values related to intrinsic and extrinsic factors, such as the environmental conditions of the species under human care. Due to this variability, few published ranges for these species can be considered definitive and must be adapted to the particular characteristics of each individual [13]. The RI provided in this study, using a homogeneous population of clinically healthy individuals maintained under human care, establish an initial analytical benchmark for the species and contribute to the limited data available on hematology and plasma chemistry analysis in *Varanus*.

Additionally, another potential limitation was the imprecision of hematological results derived from blood samples preserved in formalin, which led to the exclusion of all counts obtained using this method, resulting in certain parameters being measured in a smaller sample size.

The ASVCP recommends a minimum of 120 individuals for non-parametric methods and a 90% confidence interval to establish reference intervals. If this number is not achieved, parametric methods with 40–120 samples are recommended to ensure reliable intervals [1]. However, this number of individuals is difficult to achieve for non-conventional species [1,2,3]; therefore, smaller sample sizes are often unavoidable for RV determination. In these cases, the ASVCP allows the use of sample sizes of between 20 and 40 individuals, including statistics such as mean, median, maximum and minimum values, and a histogram of each parameter analyzed [1,2,3], as has been described in our study. The species in our study are difficult to access because, despite their increasing presence in zoological and private collections, they are still scarce and there are usually no populations of more than 5–10 individuals in a single city or even country [38]. It should be noted that the zoological collection from which the samples were taken contains the largest number of individuals of these species in Spain. In this study, the decision to unify the three species was supported by statistical analysis (Appendix A.3), which confirmed that no individual values significantly altered the studied parameters. Consequently, the results for *Varanus macraei*, *Varanus prasinus*, and *Varanus beccarii* were combined to enhance sample size and establish more representative reference intervals [19]. This approach is further justified by the close genetic relationship among these species. Notably, *Varanus beccarii* was considered a subspecies of *Varanus prasinus* throughout most of the 20th century [19]. It was only towards the end of the century that these subspecies were elevated to full species status. Additionally, *Varanus macraei* was described later as a distinct species from the time of its discovery. Given their shared evolutionary history and genetic similarities, their unification in this study provides a meaningful and practical framework for establishing reference values.

### 4.1. Hematology

Manual blood cell counting remains the most commonly used method in reptile clinical practice, despite an estimated margin of error of 10–20% [14]. This preference arises primarily from the limitations of automated hematology analyzers, which struggle to accurately differentiate blood cells in reptiles due to their unique morphology. Unlike mammals, reptilian erythrocytes and platelets are nucleated and share similar sizes with small lymphocytes and other leukocytes, making them difficult to distinguish using electronic counters that rely on electrical impedance [14]. As a result, manual counting remains the most reliable approach for obtaining accurate hematological data in these species [14]. In view of the results of the pilot study using formalin as a preservative, which showed a progressive decrease in cell count accompanied by signs of cell degradation, we could indicate that using this method would not be suitable for the preservation of blood samples from monitor lizards. This finding stands in contrast to studies involving fish, particularly elasmobranchs, where formalin has been shown to effectively preserve blood cells [34,35]. The discrepancy in results suggests that formalin may not be a suitable preservative for reptilian blood cells, potentially due to physiological differences between reptiles and elasmobranchs. One possible explanation for this difference lies in the distinct plasma osmolarity of reptiles compared to elasmobranchs. Reptiles typically have lower plasma osmolarity, which may render their cells more susceptible to damage from formalin. Additionally, the Natt and Herrick solution used in this study contains formalin, and it is possible that the concentration of formalin in our protocol was not optimized for reptilian blood, leading to overestimation or excessive exposure. This could have contributed to the observed degradation of cells over time. These findings highlight the need for further research to identify alternative preservation methods that are better suited for reptilian blood cells. Future studies could explore the use of different fixatives or adjust the concentration of formalin to better match the physiological conditions of reptiles. Understanding the specific requirements for preserving reptilian blood cells is crucial for advancing hematological research in these species, as well as for improving diagnostic and conservation efforts.

The classification of leukocytes in reptiles is complex due to morphological variation between species and the diversity of nomenclature in the scientific literature [39,40]. In addition, cell counts and percentages vary widely even in healthy individuals of the same species, influenced by physiological factors such as season, temperature, sex, reproductive status and age [14].

In this study, the application of two distinct staining methods provided an opportunity to compare the quality of cell visualization and the differentiation of various cell types in reptilian blood samples. While no significant differences were observed between the two methods, each staining technique demonstrated unique advantages and limitations that are worth considering for future research and diagnostic applications. Diff-Quik represents a method for quick staining that proves beneficial for fast result delivery in busy clinical environments. These staining techniques demonstrate various unsatisfactory points, especially when used for the analysis of reptilian blood cells. The Diff-Quik staining technique induces harmful effects on lymphocytes, which lead to unclear leukocyte differentiation particularly when observing heterophils and basophils. The potential fusion of granules and degranulation processes during analysis create difficulties in identifying important cellular features and complicate microscopic assessment [14,39]. The interpretation of rapid stain results should be done with care because these techniques create problems when analyzing leukocytes, particularly in species with challenging leukocyte identification. In contrast, May-Grünwald-Giemsa staining provides superior leukocyte identification, as well as platelet and immature erythrocyte differentiation, even though it requires a longer preparation time. The use of this staining method enables better cell morphology evaluation that remains vital for accurate reptilian blood analysis. Research and clinical applications benefit from May-Grünwald-Giemsa staining because this method enables investigators to detect faint cellular variations [14]. Researchers should investigate how to enhance reptile blood staining protocols in order to merge fast staining techniques with precise detailed methods that promote both quick results and accurate diagnoses.

Reptile leukocytes are divided into granulocytes (heterophils, eosinophils and basophils) and mononuclear leukocytes (lymphocytes and monocytes). Azurophil is a cell frequently described in the reptile veterinary literature, but its classification and identification have been controversial [41,42]. Although it has been proposed as a granulocyte, neutrophil or monocyte, its granulopoietic origin is not documented. Morphologically, it resembles monocytes with azurophilic granules occasionally observed in the peripheral blood of mammals and birds, and ultrastructurally and cytochemically, it shares similarities with monocytes [14]. Since their lineage remains unestablished and their differentiation from monocytes lacks clinical utility in reptiles, in this study, azurophils have been included within monocytes.

In our study, all cell types except eosinophils were observed. The high predominance of lymphocytes can be easily observed, with a much higher percentage than in other species of monitor lizards, such as *Varanus varius* or *Varanus exanthematicus*, and other reptile species, such as Heloderma [6,7,8]. The absence of eosinophils may be possibly due to the characteristics of species in the Toxicofera clade, which often lack eosinophils [14,40]. In addition, this may be related to the type of stains used in this study, the lack of immunohistochemical stains that would actually determine them, and the use of heparin as an anticoagulant instead of ethylenediaminetetraacetic acid (EDTA) [43]. Another factor that could explain the absence of eosinophils in our study is the specific biological characteristics of the species examined, which belong to the Toxicofera clade. Previous research has indicated that different species within this clade, including V*aranus salvator*, lack eosinophils [14,32]. The role and presence of eosinophils in reptiles remain poorly understood, adding complexity to the interpretation of hematological data in these species [14]. The occurrence of eosinophils in reptiles is highly variable, and their identification primarily relies on morphological characteristics. However, even within reptiles, there is significant heterogeneity in the staining properties and features of these cells. For example, some reptilian eosinophils exhibit benzidine peroxidase positivity, a trait commonly associated with mammalian eosinophils, while others, such as those in the green iguana (*Iguana iguana*), lack this characteristic entirely. Despite their shared nomenclature with mammalian eosinophils, there is no evidence to support a similar immunological role in reptiles; therefore, further research is needed to define the evolutionary and functional significance of these cells in reptiles [44,45].

The interpretation of the leukogram is complex, and its diagnostic value depends on the animals being kept under conditions similar to those used in the study. The leukocyte response to infection in reptiles is poorly understood, and although a count above 30,000 cells/µL is suggestive of inflammation, changes may manifest as cell ratios or cytotoxicity rather than overt leukocytosis [11]. Furthermore, it is also important to consider the potential influence of stress on leukocyte profiles when interpreting hematological data in reptiles. Stress has been shown to significantly alter leukograms, often leading to an increase in heterophils and a corresponding decrease in lymphocytes, a phenomenon known as the heterophil-to-lymphocyte (H:L) ratio shift. This stress-induced leukocyte response has been documented in various species, including reptiles, and can complicate the interpretation of hematological results [46]. As the immune response in reptiles is less predictable than in other vertebrates, an accurate diagnosis requires analysis in conjunction with microbiological studies, as well as environmental and clinical data [15]. Despite its limitations, the leukogram remains relevant, especially in monitor lizards, where cutaneous or subcutaneous bacterial infections are common [16].

Hematological tests are therefore useful tools for the diagnosis and management of diseases such as anemia, infectious diseases and others, as long as these factors are taken into account to avoid misinterpretation [14]. As previously mentioned, the interpretation of hematological values in reptiles requires consideration of several intrinsic and extrinsic factors that can significantly influence these parameters. Our study did not find statistically significant differences between sexes in any of the hematological parameters. However, other studies in reptiles such as the forest hinge-back tortoise (*Kinixys erosa*) or the grass snake (*Natrix natrix*) have reported higher hematocrit values in males compared with females [14]. These higher erythrocyte counts in males may be related to increased testosterone levels, as this hormone has been shown to play an important role in increasing erythropoiesis in various species, including reptiles [47]. In addition, males exhibit more active behavior during the breeding season, which implies higher energy expenditure and increased basal metabolism. These physiological demands could justify higher hematocrit and hemoglobin levels. However, the lack of statistical significance in our study may indicate more moderate testosterone levels in captive males. It also highlights the need to further investigate these patterns with larger samples to provide more power to the statistical analysis.

The comparison of the RVs obtained in this study with other species is complex due to the lack of methodological standardization in hematological analyses of different studies in similar species; it may also be due to the different environmental conditions, or simply due to some variability between closely related species. Despite this, there is relative agreement with the ranges reported in other phylogenetically related species from Southeast Asia (*V. salvator* or *V. komodoensis*) [48] for some parameters (hematocrit, hemoglobin, leucocyte count and cell percentages), which supports the validity of the results obtained in our study and their clinical usefulness. However, other lizards from North America that do not belong to the family Varanidae but belong to the suborder Anguimorpha (*Heloderma* spp.), such as the monitor lizards, predictably show fewer similarities [5,6,7,8,31,32,33] (Table 6).

### 4.2. Biochemistry

Biochemical tests are essential tools for diagnosing and monitoring diseases in reptiles, with some of the most commonly used and well-understood parameters including total protein, glucose, uric acid, aspartate aminotransferase (AST), calcium and phosphorus [9]. A complete biochemical profile provides a comprehensive assessment of key physiological processes, abnormalities in organ function, metabolic imbalances, and systemic health. Previous studies on biochemical parameters in related *Varanus* species, such as *Varanus salvator* and *Varanus komodoensis*, have provided some baseline data for comparison [30,33]. When comparing our results with these studies, we found similarities in several key parameters, including uric acid, total protein, albumin, calcium, phosphorus, and magnesium. These similarities suggest that certain biochemical values may be relatively preserved across closely related species within the *Varanus* genus. However, despite this agreement, significant variability exists, particularly for parameters such as liver enzymes (AST, ALT), creatine kinase, amylase, and glucose. These differences may arise from variations in methodologies, environmental conditions, seasonal changes, and intrinsic factors such as reproductive status, age, or natural variability between closely related species [5,6,7,8,9,31,32,33].

For instance, the uric acid levels observed in our study (0.0–12.2 mg/dL) align with those reported for *Varanus salvator* (1.0–12.2 mg/dL) and *Varanus komodoensis* (1.0–15.8 mg/dL) [30,33]. Uric acid is the primary catabolic product of nitrogen in reptiles. Hyperuricemia, often associated with kidney disease, has limited diagnostic sensitivity and specificity; it may be due to nephritis, nephrotoxicity or uric gout, but also to a high-protein diet or severe dehydration [9]. Uric gout, which is common in monitor lizards, is associated with poor uric acid excretion, usually related to chronic dehydration due to inadequate environmental conditions, producing the accumulation of uric acid in the tissues [16,49]. Similarly, the calcium and phosphorus levels in our study (8.6–14.9 mg/dL and 3.3–8.8 mg/dL, respectively) align with those reported for other *Varanus* species, such as *Varanus exanthematicus* (10.8–16.5 mg/dL and 0.8–7.7 mg/dL) [6]. The measurement of calcium and phosphorus levels can help detect imbalances in these minerals that can induce secondary nutritional hyperparathyroidism, a condition that leads to the development of metabolic bone disease, with signs including fibrous osteodystrophy and pathological fractures [9]. In reptiles kept in inadequate conditions, especially juveniles, hypocalcemia is often observed in association with calcium deficiency, phosphorus excess, vitamin D3 deficiency or inadequate exposure to ultraviolet B light [16] (Table 7).

On the other hand, AST and ALT liver enzymes exhibited wider ranges in our study compared to other species, possibly because of the diet, activity level or specific physiological adaptations of the *Hapturosaurus* subgenus.

Additionally, in terms of the sex-related differences found in some parameters, the significantly higher cholesterol (females—154.167 ± 48.805 mg/dL; males—76.473 ± 24.051 mg/dL) and triglyceride (females—181.034, IQR 69.3–196.2 mg/dL; males—58.225, IQR 54.4–136.1 mg/dL) levels seen in females in our study may be attributable to reproductive function. Reptiles are known to alter the lipid metabolism during vitellogenesis, with reproductive females often showing higher levels of these parameters because of egg production demands [9,50]. The assessment of reproductive function in female monitor lizards is essential to anticipating common problems such as egg retention, which is usually associated with stress or the lack of a suitable laying site [16]. During vitellogenesis and egg production, hypercholesterolemia, hypertriglyceridemia, hypercalcemia, hyperphosphatemia and hyperproteinemia with hyperglobulinemia and hyperalbuminemia are common [11,14].

In our study, statistically significant differences were also found between males and females for bile acids (females—33.671 ± 19.537; males—17.078 ± 14.369 µmol/L), with females exhibiting higher levels. This is interesting, as although sex-related variations in bile acid levels have been previously reported in other reptile species, such as *Testudo hermanni*, the direction of this difference appears to be species-specific. Leineweber et al. (2019) found significantly higher bile acid concentrations in males compared to females in *T. hermanni*, particularly during the spring [51]. These molecules play an important role in lipid, glucose and energy homeostasis, and are the main pathway for cholesterol catabolism [52]. In contrast, our results suggest that in *Hapturosaurus* spp., bile acid levels are elevated in females, which may be associated with their reproductive physiology, including vitellogenesis and lipid metabolism during the breeding season, as the increase in triglycerides and cholesterol during this period could also favor an increase in bile acid levels [9]. These interspecific differences underscore the need for species-specific reference intervals and further investigation into the physiological and endocrine factors that influence bile acid dynamics in reptiles.

When comparing liver enzyme values, such as AST, ALT, and GGT, with those of other species, we observed some variability. AST levels in our study (0.0–85.6 U/L) were within the range reported for *Varanus salvator* (2.0–58.0 U/L) and *Varanus exanthematicus* (5.0–80.0 U/L) [6,33]. However, ALT levels in our study (<8 U/L) were lower than those reported for *Varanus exanthematicus* (7.0–374.0 U/L), which may reflect differences in liver metabolism or dietary influences [9]. GGT levels in our study (0.0–5.8 U/L) were consistent with the minimal activity typically observed in reptiles, supporting the notion that GGT has limited diagnostic utility in these species [9,11]. In reptiles, AST is one of the most important enzymes in the biochemistry panel due to its high activity in liver tissue, although its presence in other tissues, such as muscle and heart, makes it less specific for liver disease compared to other animals. Therefore, elevated AST levels in reptiles must always be interpreted alongside CK to differentiate between hepatic and muscular pathologies. In cases where both enzymes are elevated, muscle injury should be prioritized as a differential diagnosis, whereas isolated AST elevation may suggest hepatic involvement [11]. Other enzymes, such as ALT and ALP, may be elevated in cases of lipidosis or liver degeneration, but they are also not specific to the liver [9,11]. While glutamate dehydrogenase (GLDH) is a liver-specific enzyme in mammals, its diagnostic application in most reptiles, including *Hapturosaurus* species, remains unexplored. Future studies should investigate GLDH activity to determine its potential as a more specific marker for hepatic injury in these lizards.

The wide range of glucose levels observed in our study (73.5–171.7 mg/dL) could be influenced by factors such as stress, fasting, or even the time from feeding to blood collection [16]. It is also important to note that methodological factors, such as animal handling, sample collection techniques, and the time elapsed from sample collection to processing, can significantly influence biochemical results. For example, glucose levels are particularly sensitive to delays in processing, as they tend to decrease over time due to ongoing glycolysis in the blood sample [9]. This highlights the importance of carefully recording and describing all steps in the sampling process, including the time from collection to analysis, to ensure the accuracy and reliability of the results. In our study, samples were processed within a maximum of 120 minutes after collection to minimize such effects, but even small variations in handling or processing times could introduce variability in the results [53].

### 4.3. Main Limitations

A limitation to our study regarding albumin is that a laboratory-based chemical analyzer was used for its analysis. Previous studies have described in other saurian species that values obtained by laboratory-based chemical analyzers using the dye bromocresol green (BCG) to quantify albumin are remarkably high compared to those obtained by protein electrophoresis [54]. Future studies using both techniques to measure albumin in these species would be of interest.

Another significant limitation of this study is the small sample size, which, while sufficient for establishing preliminary reference intervals, may not fully capture the true natural variability within these *Varanus* species. This limitation is largely due to the inherent challenges of working with these reptiles, including their limited availability in captivity, the difficulty of obtaining blood samples from healthy homogeneous populations, and the need to minimize stress during handling and sampling. According to the ASVCP, the sample size should ideally be between 40 and 120 individuals for all hematological and biochemical parameters so as to achieve a more reliable clinical application [1]. Therefore, future studies should aim to increase sample sizes by collaborating with other zoos and private collections that house monitor lizards of these species under similar environmental conditions. Additionally, standardizing blood collection and analysis methodologies will be crucial for ensuring consistency and reliability in the data. Although the sample size allowed us to define initial reference intervals, it may not account for the full range of physiological variability influenced by factors such as age, sex, reproductive status, and environmental conditions. In addition, a procedural feature of this study allowed the number of individuals to be increased by grouping together three closely related species (*Varanus macraei*, *Varanus prasinus*, and *Varanus beccarii*) that are currently considered distinct for the purpose of establishing the RV. Ideally, separate RVs should have been determined for each species. Due to their rarity and phylogenetic similarity, they were grouped together. Statistical analyses confirmed that there were no significant differences between them, thus supporting this approach. However, future studies should aim to establish species-specific reference intervals to account for possible physiologic differences [9].

### 4.4. Conclusions

Taking into consideration the above limitations for the present study, future research will need to expand sample size through work with other zoological institutions and private collections that also maintain the species under equivalent environmental conditions. In addition, the use of standardized protocols (as described in this manuscript) for sample collection, handling and processing will reduce variability and will provide consistency between studies. Investigation into the biochemistry of the intrinsic factors such as reproductive status, age, and sex will further provide a better understanding of physiological variability within these species. So, by addressing the limitations of this study and expanding the scope of future research, we can improve the diagnostic tools available for these species and contribute to their effective clinical management and conservation.

## 5. Conclusions

In conclusion, the hematological and plasma chemistry data obtained in the present study could be a valuable tool for assessing the health status of V*aranus macraei*, *Varanus prasinus*, and *Varanus beccarii* under human care, particularly under the environmental conditions described. These findings not only contribute to a better understanding of the blood parameters specific to these species, but also enhance our knowledge of the subgenus *Hapturosaurus*, for which hematological data are currently limited. The results of this study have practical implications for veterinarians and caretakers working with *Varanus* species, as they support the development of effective health monitoring, diagnostic techniques and management programs, as well as informed breeding strategies. By filling gaps in the existing knowledge, this research contributes to promoting the care and well-being of *Varanus* species, as well as supporting the conservation efforts aimed at preserving these unique and ecologically important reptiles.

## Figures and Tables

**Table 1:** Results of the hematological study in monitor lizards of the subgenus *Hapturosaurus* (pool of individuals of *V. macraei*, *V. prasinus*, *V. beccarii* of both sexes).

	n	Mean	Median	SD	Minimum	Maximum	RI	LRL	URL
HTC (%)	32	35.3	34.8	4.3	26.5	45.0	26.4–44.2	24.5–28.3	43.2–45.8
Hb (g/dL)	17	9.8	9.7	0.9	8.5	11.9	7.7–11.9 *	7.1–8.4	11.2–12.7
RBC (×10^6^/µL)	18	1.09	1.1	0.03	0.48	1.63	0.36–1.82	0.30–0.41	1.73–1.92
MCV (fL)	17	357.9	311.0	127.5	214.7	618.6	189.5–776.7 *	160.0–219.0	745.0–810.0
MCH (pg)	16	93.9	87.8	23.9	65.6	142.6	41.4–146.4 *	36.0–46.0	140.0–152.0
MCHC (g/dL)	17	28.2	28.6	2.7	22.1	32.9	22.3–34.1 *	20.0–24.0	33.0–35.6
WBC (×10^3^/µL)	15	6.2	6.6	2.2	1.4	9.5	1.2–11.2 *	0.9–1.5	10.5–12.0
Heterophils (%)	32	38.2	38.0	8.4	20.0	54.0	20.9–55.5	18.5–20.9	55.5–58.2
Lymphocytes (%)	32	55.0	53.8	7.7	38.0	71.0	39.1–70.9	36.9–39.1	70.9–73.2
Monocytes (%)	32	6.7	7.0	3.3	1.5	14.0	0.0–13.6	0.0–0.5	13.6–14.5
Basophils (%)	34	0.1	0.0	0.3	0.0	1.0	<1.0	0.0–0.0	1.0–1.1

SD: Standard deviation. RI: Reference interval. HCT: Hematocrit. Hb: Hemoglobin. RBC: Red blood cell count. MCV: Mean corpuscular volume. MCH: Mean corpuscular hemoglobin. MCHC: Mean corpuscular hemoglobin concentration. WBC: Total white blood cell count. The asterisk (*) indicates that the sample size (*n*) was less than 20; therefore, the reference intervals should be interpreted with caution, as recommended by the ASVCP.

**Table 2:** Statistical analysis (Student’s *t*-test). Comparison between staining methods used for leukocyte differential counts (*Diff-Quik* and *May-Grünwald Giemsa*).

Parameter	Mean		SD		Homogeneity of Variances Test	*t*-Test for Equality of Means
	Diff-Quik (*n =* 43)	Giemsa (*n* = 20)	Diff-Quik	Giemsa	Sig.	Two-Tailed *p*-Value (Sig.)
Heterophils (%)	38.9	36.4	11.7	10.9	0.6	0.4
Lymphocytes (%)	54.1	55.8	10.5	8.7	0.2	0.5
Monocytes (%) *	6.9	7.5	4.3	6.2	0.5	0.6
Basophils (%)	0.1	0.2	0.2	0.5	0.0	0.2
Eosinophils (%)	No detected	No detected	-	-	-	-

Sig: Significance. * Azurophils were included within the monocyte count when observed.

**Table 3:** Statistical analysis (Student’s *t*-test). Comparison between sexes for hematological parameters.

Parameter	n		Male			Female		Homogeneity of Variances Test	*t*-Test for Equality of Means
		N	Mean	SD	N	Mean	SD	Sig.	Two-Tailed
									*p*-Value (Sig.)
HTC (%)	32	16	36.5	5.9	16	34.3	4.5	0.198	0.242
Hb (g/dL)	17	9	10.4	1.7	8	9.6	0.7	0.060	0.207
RBC (×10^6^/µL)	18	9	1.0	3.3	9	1.1	3.5	0.539	0.507
MCV (fL)	17	9	387.7	1.2	8	3.3	1.3	0.712	0.381
MCH (pg)	16	8	110.1	3.7	8	89.9	25.8	0.457	0.210
MCHC (g/dL)	16	8	28.4	2.0	8	28.1	3.3	0.217	0.799
WBC (×10^3^/µL)	19	10	6.5	1.6	9	9.8	6.9	0.005	0.209
Heterophils (%)	18	9	39.5	7.5	9	40.5	13.4	0.096	0.791
Lymphocytes (%)	18	9	54.8	7.3	9	52.2	11.6	0.168	0.462
Monocytes (%)	17	9	5.6	2.7	8	7.2	3.9	0.245	0.205
Basophils (%)	16	8	0.1	0.3	8	0.9	0.3	0.827	0.909

HTC: Hematocrit. Hb: Hemoglobin. RBC: Red blood cell count. MCV: Mean corpuscular volume. MCH: Mean corpuscular hemoglobin. MCHC: Mean corpuscular hemoglobin concentration. WBC: Total white blood cell count. SD: Standard derivation. Sig: Significance.

**Table 4:** Results of the blood biochemistry study in monitor lizards of the subgenus Hapturosaurus (pool of individuals of *V. macraei*, *V. prasinus*, *V. beccarii* of both sexes).

	n	Mean	Median	SD/IQR	Min	Max	RI	LRL	URL
ALT (U/L)	31	2.5	2.0	2.2	0.0	8.0	<8.0	0.0–0.4	7.6–8.0
ALP (U/L)	32	479.2	485.8	138.5	186.9	760.9	192.4–765.9	170.0–192.4	765.9–781.0
GGT (U/L)	30	2.4	1.9	1.7	0.3	6.5	0.0–5.8	0.0–0.2	5.8–6.7
AST (U/L)	32	39.7	36.3	22.2	7.8	99.1	0.0–85.6	0.0–4.0	85.6–93.0
CK (U/L) *	32	-	1797.1	943.3–1865.0	188.3	5877.9	0.0–5218.6	0.0–275.0	5218.6–5680.0
Bile Acids (µmol/L)	33	25.6	23.4	18.9	1.5	72.8	0.0–64.8	0.0–5.0	64.8–73.5
Urea (mg/dL)	31	4.9	4.7	1.2	2.6	7.3	2.3–7.5	2.0–2.5	7.5–8.0
Uric Acid (mg/dL) *	33	-	5.1	3.9–7.7	1.6	13.8	0.0–12.2	0.0–1.6	12.2–13.8
Total Solids (g/dL)	34	7.3	7.2	0.8	6.0	9.7	5.6–8.9	5.6–6.0	8.9–9.7
Total Proteins (g/dL)	33	6.5	6.5	0.9	4.7	8.2	4.6–8.4	4.4–4.9	7.9–8.2
Albumin (g/dL)	33	2.7	2.7	0.4	2.0	3.4	1.9–3.5	1.9–2.0	3.0–3.4
Glucose (mg/dL)	33	122.6	118.4	23.7	74.0	179.0	73.5–171.7	73.5–77.5	171.7–179.0
Fructosamine (µmol/L)	31	184.4	183.2	30.5	123.1	236.3	121.0–247.7	121.0–126.0	234.3–247.7
Lactate (mg/dL)	32	44.7	44.8	16.1	21.6	82.5	11.3–78.1	11.3–14.1	78.1–82.9
Cholesterol (mg/dL)	33	118.9	111.4	55.3	41.6	245.6	4.4–233.3	73.4–80.2	233.0–245.6
Triglycerides (mg/dL) *	25	-	43.5	41.2–126.8	9.8	168.7	0.0–137.3	4.4–9.2	137.3–146.2
Amylase (U/L)	30	1256.5	1266.4	522.2	412.9	2543.2	170.8–2342.2	170.8–188.0	2342.2–2543.0
Calcium (mg/dL)	33	11.7	11.7	1.5	9.2	14.7	8.6–14.9	8.6–9.2	14.9–15.3
Phosphorus (mg/dL)	29	6.0	6.0	1.3	3.8	8.8	3.3–8.8	3.3–3.6	8.8–9.1
Ca:P	33	1.9	1.9	0.6	0.8	3.9	0.6–3.2	1.6–1.8	3.2–3.5
Magnesium (mg/dL)	33	2.5	2.5	0.3	1.9	2.9	1.9–3.0	1.9–2.0	3.0–3.2

SD: Standard deviation. IQR: interquartile range. Min: minimum. Max: maximum. RI: reference interval. LRL, 90% confidence interval of the lower reference limit; URL, 90% confidence interval of the upper reference limit. ALT: Alanine aminotransferase. ALP: Alkaline phosphatase. GGT: Gamma-Glutamyl Transferase. AST: Aspartate aminotransferase. CK: Creatine kinase. Ca:P: Calcium-to-phosphorus ratio. Asterisk denotes the use of the median in place of the mean, the range instead of the standard deviation, and the application of the Kruskal–Wallis test as an alternative to the Student’s *t*-test due to non-normal data distribution. The asterisk (*) indicates that the parameters were analyzed using a robust parametric method.

**Table 5:** Statistical analysis (Student’s *t*-test o Kruskal–Wallis test). Comparison between sexes for blood biochemistry parameters.

Parameter	Male (*n =* 16)		Female (*n =* 18)		Homogeneity of Variances Test	*t*-Test o Kruskal–Wallis Tests
	Mean/Median	SD/IQR	Mean	SD/IQR	Sig.	Two-Tailed *p*-Value (Sig.)
ALT (U/L)	3.4	4.3	2.6	2.2	0.241	0.536
ALP (U/L)	469.2	126.1	462.2	183.1	0.379	0.902
GGT (U/L)	2.8	2.4	3.7	4.4	0.197	0.486
AST (U/L)	47.3	35.8	39.7	23.4	0.430	0.472
CK * (U/L)	2816.4	923.6–1895.7	2097.8	933.3–1886.1	0.312	0.324
Bile Acids (µmol/L)	17.1	14.4	33.6	19.5	0.226	0.010
Urea (mg/dL)	4.9	1.4	4.7	1.5	0.473	0.775
Uric Acid (mg/dL) *	4.9	3.6–7.2	8.1	3.7–8.5	0.091	0.081
Total Solids (g/dL)	7.1	0.7	7.4	0.8	0.953	0.166
Total Proteins (g/dL)	6.4	0.8	6.5	0.9	0.609	0.645
Glucose (mg/dL)	129.1	23.1	117.1	23.5	0.912	0.151
Albumin (g/dL)	2.7	0.4	2.6	0.4	0.948	0.689
Fructosamine (µmol/L)	184.0	35.3	175.4	45.9	0.961	0.567
Lactate (mg/dL)	41.3	15.5	47.7	16.5	0.692	0.268
Cholesterol (mg/dL)	76.5	24.1	154.1	48.8	0.007	<0.001
Triglycerides (mg/dL) *	58.2	54.4–136.1	181.0	69.3–196.2	0.001	0.016
Amylase (U/L)	1440.2	696.4	1409.5	796.8	0.940	0.908
Calcium (mg/dL)	11.7	1.4	11.7	1.6	0.573	0.932
Phosphorus (mg/dL)	6.1	1.3	7.4	3.1	0.009	0.121
Magnesium (mg/dL)	2.4	0.3	2.5	0.2	0.864	0.172

ALT: Alanine aminotransferase. ALP: Alkaline phosphatase. GGT: Gamma-Glutamyl Transferase. AST: Aspartate aminotransferase. CK: Creatine kinase. SD: Standard derivation. IQR: interquartile range. Sig: Significance. Asterisk denotes the use of the median in place of the mean, the range instead of the standard deviation, and the application of the Kruskal–Wallis test as an alternative to the Student’s *t*-test due to non-normal data distribution. The asterisk (*) indicates that the parameters were analyzed using a robust parametric method.

**Table 6:** Reference values of hematology parameters for different species of anguimorph reptiles. Data for this work are shown in bold in the last row.

Species	HCT (%)	Hb (g/dL)	RBC (×10^6^/mm^3^)	WBC (×10^3^/mm^3^)	Heterophils (%)	Lymphocytes (%)	Monocytes (%)	Basophils (%)	References
*Varanus salvator*	28–36	8.7–12.4	0.9–1.3	9.9–21.9	27.5–50.7	32.1–54.9	11.0–24.8	0.5–1.1	[32]
*Varanus niloticus*	24–31	6.9–12.5	0.6–1.7	1.6–2.3	13.0–34.0	58.0–74.0	6.0–13.0	0.0–2.0	[31]
*Varanus varius*	29–43	-	-	0.6–63.8	2.0–68.0	12.0–81.0	7.0–57.0	0.0	[8]
*Varanus komodoensis*	25–40	9.5–15.9	-	0.7–12.5	8.0–58.0	35.0–82.0	0.0–23.0	0.0–13.0	[30]
*Varanus exanthematicus*	16–51	6.2–13.2	0.6–1.6	0.1–10.9	1.0–45.5	1.0–49.0	0.0–23.0	0.0–3.0	[33]
*Heloderma suspectum*	32–44	6.5–8.0	0.4–0.6	4.2–5.2	40.0–49.0	17.0–39.0	3.0–12.0	10.0–12.0	[6]
*Heloderma horridum*	28–41	7.0–10.9	0.7–1.1	2.9–7.3	48.0–61.0	11.0–16.0	7.0–33.0	1.0–2.0	[7]
***Hapturosaurus*** **spp.**	**26–44**	**7.7–11.9**	**0.4–1.8**	**1.2–11.2**	**20.9–55.5**	**39.1–70.9**	**0.0–13.6**	**<1.0**	**-**

HCT: Hematocrit. Hb: Hemoglobin. RBC: Red blood cell count. WBC: Total white blood cell count.

**Table 7:** Reference values of blood biochemistry parameters for different species of anguimorph reptiles. Data for this work are shown in bold in the last row.

Species	ALT (U/L)	ALP (U/L)	GGT (U/L)	AST (U/L)	CK (U/L)	Chol (mg/dL)	Amylase (U/L)	References
*Varanus salvator*	1.0–93.0	14.0–405.0	7.0–48.0	2.0–58.0	176.0–1818.0	22.0–126.0	265.0–1868.0	[33]
*Varanus exanthematicus*	7.0–374.0	4.0–101.0	1.0–11.0	5.0–80.0	7.0–6624.0	49.0–231.0	-	[33]
*Varanus varius*	-	-	-	11.6–36.0	50.0–809.0	-	-	[8]
*Varanus dumerilii*	-	-	-	48.7	-	178.6	1997.0	[5]
*Varanus komodoensis*	-	-	-	7.0–39.0	130.0–2403.0	-	-	[30]
*Heloderma suspectum*	-	-	-	30.0–51.0	253.0–830.0	-	-	[6]
*Heloderma horridum*	10.0–25.0	7.0–47.0	0.0–16.0	13.0–50.0	43.0–107.0	-	123.4–3673.0	[7]
***Hapturosaurus* spp.**	**<8.0**	**192.0–766.0**	**0.0–5.8**	**0.0–85.6**	**0.0–5218.6**	**4.4–233.3**	**170.8–2342.2**	**-**
**Species**	**AU (mg/dL)**	**TP (g/dL)**	**Alb (g/dL)**	**Glu (mg/dL)**	**Ca (mg/dL)**	**P (mg/dL)**	**Mg (mg/dL)**	**References**
*Varanus salvator*	1.0–12.2	5.1–9.8	1.4–3.4	29.0–170.0	9.8–18.2	2.9–8.9	2.2–2.7	[33]
*Varanus exanthematicus*	2.0–14.6	3.4–9.8	0.6–3.3	54.0–163.0	10.8–16.5	0.8–7.7	3.1	[33]
*Varanus varius*	2.0–15.5	5.8–9.6	1.8–3.5	90.0–329.0	-	2.5–8.6	-	[8]
*Varanus dumerilii*	3.5	7.1	2.7	81.9	16.3	4.9	-	[5]
*Varanus komodoensis*	1.0–15.8	5.1–9.9	3.0–4.5	121.0–259.0	12.0–16.8	2.3–8.7	2.5–4.1	[30]
*Heloderma suspectum*	14.1–19.0	6.1–6.6	-	36.0–59.0	11.7–13.0	2.3–4.0	-	[6]
*Heloderma horridum*	1.1–7.4	3.2–7.5	0.9–1.4	50.0–117.0	9.4–18.2	2.4–4.9	3.1–3.5	[7]
***Hapturosaurus* spp.**	**0.0–12.2**	**4.6–8.4**	**1.9–3.5**	**73.5–171.7**	**8.6–14.9**	**3.3–8.8**	**1.9–3.0**	**-**

ALT: Alanine aminotransferase. ALP: Alkaline phosphatase. GGT: Gamma-Glutamyl Transferase. AST: Aspartate aminotransferase; CK. Creatine kinase. Chol: Cholesterol. AU: Uric acid. TP: Total proteins. Alb: Albumin. Glu: Glucose. Ca: Calcium. P: Phosphorus. Mg: Magnesium.

## Data Availability

All data will be available upon request to the authors.

## Abbreviations

The following abbreviations are used in this manuscript:

ASVCP	American Society for Veterinary Clinical Pathology
RV	Reference values
RI	Reference intervals
AST	Aspartate aminotransferase
IUCN	International Union for Conservation of Nature
PCV	Manual packed cell volume
WBC	White blood cell
HTC	Hematocrit
HB	Hemoglobin
RBC	Red blood cells
MCV	Mean corpuscular volume
CHCM	Concentración de hemoglobina corpuscular media
CK	Creatinin kinase
ALT	Alanine aminotransferase
GGT	Gamma-glutamyltransferase
TS	Total solids
EDTA	Ethylenediaminetetraacetic acid

## Appendix A

### Appendix A.1

**Table A1:** Individuals (*n* = 34) used in the study. Identification, species, sex, and morphometric parameters.

Microchip	Species	Sex	Weight (g)	TL (cm)	SVL (cm)
704	*V. macraei*	Female	247	88.0	33.0
1111	*V. macraei*	Female	255	83.5	28.0
1733	*V. macraei*	Female	345	94.0	30.0
2024	*V. macraei*	Female	204	78.0	29.0
2031	*V. macraei*	Female	210	77.0	27.0
2039	*V. macraei*	Female	205	65.0	27.0
2040	*V. macraei*	Female	227	85.0	30.0
5549	*V. macraei*	Female	235	86.0	30.0
6691	*V. macraei*	Female	148	78.0	27.0
6735	*V. macraei*	Female	247	83.5	27.5
7934	*V. macraei*	Female	277	89.0	31.0
1157	*V. macraei*	Male	388	94.0	31.0
2035	*V. macraei*	Male	239	85.0	29.0
2303	*V. macraei*	Male	382	67.0	31.0
5551	*V. macraei*	Male	361	90.0	28.0
7459	*V. macraei*	Male	248	82.0	29.0
7967	*V. macraei*	Male	530	106.0	35.0
2439	*V. prasinus*	Female	111	62.0	23.0
5405	*V. prasinus*	Female	124	61.0	23.0
5548	*V. prasinus*	Female	249	70.0	28.0
7334	*V. prasinus*	Female	168	70.0	26.0
7399	*V. prasinus*	Female	167	73.5	26.0
2437	*V. prasinus*	Male	146	67.0	26.0
2648	*V. prasinus*	Male	259	79.0	27.0
2874	*V. prasinus*	Male	274	76.5	27.5
4297	*V. prasinus*	Male	186	73.0	26.0
5207	*V. prasinus*	Male	154	70.0	24.0
6839	*V. prasinus*	Male	220	78.0	27.0
7441	*V. prasinus*	Male	305	79.0	27.0
9493	*V. prasinus*	Male	123	64.0	23.0
8301	*V. beccarii*	Female	223	78.5	27.0
9569	*V. beccarii*	Female	259	79.5	28.5
5570	*V. beccarii*	Male	265	86.0	29.5
9407	*V. beccarii*	Male	298	81.0	28.0

TL: Total length. SVL: Snout–vent length.

### Appendix A.2

**Table A2:** Individual data on daytime temperature, relative humidity, and terrarium dimensions (height × width × depth).

Individual	Temperature (°C)	Relative Humidity (%)	Terrarium Dimensions (cm)
704	24–35	62.0	150 × 100 × 60
1111	24–33	57.0	120 × 120 × 70
1733	23–34	58.5	120 × 120 × 70
2024	24–36	66.0	150 × 100 × 60
2031	25–37	64.0	150 × 100 × 60
2039	27–32	70.0	150 × 100 × 60
2040	24–36	66.0	150 × 100 × 60
5549	26–30	61.5	150 × 100 × 60
6691	24–35	64.0	150 × 100 × 60
6735	24–33	66.5	150 × 100 × 60
7934	25–35	65.0	120 × 120 × 70
1157	24–34	69.0	120 × 90 × 50
2035	23–35	62.0	150 × 100 × 60
2303	24–30	68.0	150 × 100 × 60
5551	25–35	64.0	150 × 100 × 60
7459	24–33	55.0	120 × 90 × 50
7967	24–35	56.5	120 × 120 × 70
2439	24–29	67.0	150 × 100 × 60
5405	24–34	50.5	150 × 100 × 60
5548	24–29	70.0	150 × 100 × 60
7334	24–35	67.5	130 × 150 × 80
7399	23–34	66.0	130 × 150 × 80
2437	24–29	67.0	150 × 100 × 60
2648	25–33	65.0	120 × 100 × 60
2874	24–35	55.0	150 × 100 × 60
4297	24–36	53.0	150 × 100 × 60
5207	24–34	54.0	100 × 100 × 50
6839	24–30	66.0	150 × 100 × 60
7441	25–31	68.0	130 × 150 × 80
9493	24–29	70.0	150 × 100 × 60
8301	24–32	66.0	120 × 120 × 70
9569	24–35	54.0	120 × 90 × 50
5570	24–38	60.0	120 × 120 × 70
9407	25–37	55.0	100 × 90 × 50

### Appendix A.3

**Table A3:** Statistical analysis (one-way ANOVA). Comparison between species.

Parameter	Homogeneity of Variances Test	ANOVA
Hematocrit	0.075	0.406
Hemoglobin	0.417	0.529
RBC	0.484	0.112
WBC	0.489	0.211
Heterophils	0.715	0.424
Lymphocytes	0.930	0.781
Monocytes	0.590	0.060
Basophils	0.717	0.717
ALT	0.542	0.337
ALP	0.142	0.058
GGT	0.696	0.218
AST	0.610	0.432
CK	0.147	0.053
Bile Acids	0.294	0.050
Urea	0.250	0.480
Uric Acid	0.819	0.476
Total Solids	0.793	0.277
Total Proteins	0.313	0.787
Albumin	0.372	0.286
Glucose	0.133	0.714
Fructosamine	0.593	0.264
Lactate	0.457	0.175
Cholesterol	0.902	0.855
Triglycerides	0.696	0.915
Amylase	0.712	0.148
Calcium	0.076	0.590
Phosphorus	0.514	0.901
Magnesium	0.606	0.077

HCT: Hematocrit. Hb: Hemoglobin. RBC: Red blood cell count. MCV: Mean corpuscular volume. MCH: Mean corpuscular hemoglobin. MCHC: Mean corpuscular hemoglobin concentration. WBC: Total white blood cell count.

### Appendix A.4

#### Appendix A.4.1

**Table A4:** Individual results of hematological parameters.

Individual	HCT (%)	Hb (g/dL)	RBC (mm^3^)	MCV (fL)	MCH (pg)	MCHC (g/dL)	WBC (mm^3^)	Heterophils (%)	Lymphocytes (%)	Monocytes (%)	Basophils (%)
704	32.1	-	-	-	-	-	-	65.0	29.0	5.0	1.0
1111	35.0	10.7	1,630,000	214.7	65.6	30.6	14,474	38.0	54.5	7.5	0.0
1733	34.5	10.2	1,315,000	262.4	77.6	29.6	17,378	41.0	55.5	3.5	0.0
2024	32.0	-	-	-	-	-	-	31.0	60.0	9.0	0.0
2031	34.6	-	-	-	-	-	-	54.0	38.0	8.0	0.0
2039	37.5	-	-	-	-	-	-	22.0	71.0	7.0	0.0
2040	23.2	-	-	-	-	-	-	23.0	64.0	13.0	0.0
5549	37.0	-	-	-	-	-	-	36.0	50.0	14.0	0.0
6691	38.0	-	-	-	-	-	-	38.0	55.0	7.0	0.0
6735	34.5	10.2	1,535,000	224.8	66.5	29.6	9589	36.5	49.0	14.0	0.5
7934	33.5	9.8	1,195,000	280.3	82.0	29.3	5128	39.0	57.0	4.0	0.0
1157	31.4	10.1	1,230,000	255.5	82.1	32.1	9434	26.5	67.5	5.5	0.5
2035	42.0	-	-	-	-	-	-	38.0	52.0	10.0	0.0
2303	33.0	-	-	-	-	-	-	43.0	52.0	5.0	0.0
5551	33.0	-	-	-	-	-	-	42.0	53.0	5.0	0.0
7459	36.4	-	-	-	-	-	-	34.5	61.0	4.5	0.0
7967	39.5	11.3	1,055,000	374.4	107.1	28.6	6667	37.5	52.5	10.0	0.0
2439	34.0	-	-	-	-	-	-	51.0	47.0	2.0	0.0
5405	38.0	9.2	645,000	589.2	142.6	24.2	1471	34.0	58.0	8.0	0.0
5548	38.0	-	-	-	-	-	-	38.0	53.0	9.0	0.0
7334	26.5	8.7	950,000	278.6	91.6	32.9	8911	50.5	45.0	4.5	0.0
7399	43.0	9.5	877,500	490.0	108.3	22.1	22,396	72.0	28.0	0.0	0.0
2437	45.0	-	-	-	-	-	-	46.0	51.0	3.0	0.0
2648	44.0	11.9	1,415,000	311.0	84.1	27.1	7000	25.0	66.0	9.0	0.0
2874	30.0	9.1	485,000	618.6	187.6	30.3	5263	33.3	64.3	2.5	0.0
4297	36.0	9.6	676,700	532.0	141.9	26.7	7042	35.5	62.5	2.0	0.0
5207	33.0	8.6	1,095,000	301.4	78.5	26.1	7576	49.5	49.0	1.5	0.0

HCT: Hematocrit. Hb: Hemoglobin. RBC: Red blood cell count. MCV: Mean corpuscular volume. MCH: Mean corpuscular hemoglobin. MCHC: Mean corpuscular hemoglobin concentration. WBC: Total white blood cell count.

#### Appendix A.4.2

**Table A5:** Serial granulocyte count in a blood sample of *V. macraei* collected 1 March 2023 and preserved with formalin using the protocol described by Arnold at al., 2014 [34].

Date	Granulocyte Count (n/mm^3^)
1 March 2023	3125
8 March 2023	2600
15 March 2023	2375
23 March 2023	1750
29 March 2023	1265

### Appendix A.5

**Table A6:** Individual results of blood biochemistry parameters.

Individual	ALT (U/L)	ALP (U/L)	GGT (U/L)	AST (U/L)	CK (U/L)	BA (µmol/L)	Urea (mg/dL)	UA (mg/dL)	TS (g/dL)	TP (g/dL)	Alb (g/dL)
704	6	393.9	1.1	37.7	1219.2	24.2	5.0	5.8	7.2	6.8	2.7
1111	4	554.7	8.3	72.0	771.8	72.8	0.7	31.8	7.2	7.7	2.6
1733	0	689.4	18.7	58.8	1413.5	54.7	5.8	11.4	7.7	7.5	3.1
2024	4	318.7	2.3	15.9	874.1	30.0	4.7	6.3	7.0	5.8	2.5
2031	0	451.7	1.5	10.0	348.4	21.7	4.5	2.2	7.6	6.0	2.5
2039	7	368.0	4.3	27.2	1344.6	57.2	4.0	13.8	7.5	6.9	2.4
2040	0	340.3	2.5	14.7	503.8	38.8	3.0	5.9	7.1	4.7	2.2
5549	2	475.2	1.4	32.7	2011.3	52.5	3.7	4.1	7.8	6.9	2.9
6691	6	405.5	2.7	29.0	1482.9	34.7	5.7	5.8	8.0	6.5	2.8
6735	2	512.6	1.8	99.1	2354.0	30.9	6.4	7.2	8.7	8.2	3.3
7934	4	570.2	1.9	41.6	3201.9	24.8	4.1	5.0	9.7	8.1	3.2
1157	3	625.0	1.7	45.8	1374.6	12.9	5.3	3.7	6.6	6.2	2.5
2035	2	397.7	3.2	45.7	4227.2	5.8	5.8	3.2	6.7	5.3	2.3
2303	0	578.7	3.1	19.5	1718.7	40.8	4.0	8.3	6.5	5.6	2.3
5551	1	485.7	3.8	15.3	1012.5	19.0	3.9	3.6	7.2	6.7	2.9

ALT: Alanine aminotransferase. ALP: Alkaline phosphatase. GGT: Gamma-Glutamyl Transferase. AST: Aspartate aminotransferase. CK: Creatine kinase. BA: Bile acids. UA: Uric acid. TS: Total solids. TP: Total proteins. Alb: Albumin.

**Table A7:** Individual results of blood biochemistry parameters.

Individual	ALT (U/L)	ALP (U/L)	GGT (U/L)	AST (U/L)	CK (U/L)	BA (µmol/L)	Urea (mg/dL)	UA (mg/dL)	TS (g/dL)	TP (g/dL)	Alb (g/dL)
704	6	393.9	1.1	37.7	1219.2	24.2	5.0	5.8	7.2	6.8	2.7
1111	4	554.7	8.3	72.0	771.8	72.8	0.7	31.8	7.2	7.7	2.6
1733	0	689.4	18.7	58.8	1413.5	54.7	5.8	11.4	7.7	7.5	3.1
2024	4	318.7	2.3	15.9	874.1	30.0	4.7	6.3	7.0	5.8	2.5
2031	0	451.7	1.5	10.0	348.4	21.7	4.5	2.2	7.6	6.0	2.5
2039	7	368.0	4.3	27.2	1344.6	57.2	4.0	13.8	7.5	6.9	2.4
2040	0	340.3	2.5	14.7	503.8	38.8	3.0	5.9	7.1	4.7	2.2
5549	2	475.2	1.4	32.7	2011.3	52.5	3.7	4.1	7.8	6.9	2.9
6691	6	405.5	2.7	29.0	1482.9	34.7	5.7	5.8	8.0	6.5	2.8
6735	2	512.6	1.8	99.1	2354.0	30.9	6.4	7.2	8.7	8.2	3.3
7934	4	570.2	1.9	41.6	3201.9	24.8	4.1	5.0	9.7	8.1	3.2
1157	3	625.0	1.7	45.8	1374.6	12.9	5.3	3.7	6.6	6.2	2.5
2035	2	397.7	3.2	45.7	4227.2	5.8	5.8	3.2	6.7	5.3	2.3
2303	0	578.7	3.1	19.5	1718.7	40.8	4.0	8.3	6.5	5.6	2.3
5551	1	485.7	3.8	15.3	1012.5	19.0	3.9	3.6	7.2	6.7	2.9

ALT: Alanine aminotransferase. ALP: Alkaline phosphatase. GGT: Gamma-Glutamyl Transferase. AST: Aspartate aminotransferase. CK: Creatine kinase. BA: Bile acids. UA: Uric acid. TS: Total solids. TP: Total proteins. Alb: Albumin.

**Table A8:** Individual results of blood biochemistry parameters.

Individuo	Glucose (mg/dL)	Fructosamine (µmol/L)	Lactate (mg/dL)	Cholesterol (mg/dL)	Tryglicerides (mg/dL)	Amylase (U/L)	Calcium (mg/dL)	Phosphorus	Magnesium (mg/dL)
704	89.1	196.9	59.4	148	23.9	898.4	11.9	5.3	2.9
1111	167.9	27.6	79.3	123.1	729.9	1417.1	12.2	14.7	2.6
1733	125.9	165.8	40.6	60.5	215.3	1051.9	11.8	11.5	2.7
2024	135.6	171.1	23.2	107.8	32.1	952.3	11.4	6	2.3
2031	91.1	219.4	48.6	116.7	16.5	683.7	9.9	4.8	2.8
2039	111.6	143	82.5	188.6	394.7	1419.9	12.7	8.8	2.7
2040	113.4	178.4	45.7	126.3	15.1	865.7	9.2	4.9	2.2
5549	124.1	201.5	31.5	116	24	741.4	12.1	4.8	2.4
6691	134.6	202.5	25.7	220.8	18.6	710.9	11.6	7.3	2.5
6735	115.7	182.8	47.6	208.9	192	2519.1	13.4	11.4	2.6
7934	95.2	197.8	52.9	245.6	295	2730.4	14.7	6.2	2.6
1157	138.6	170	65.4	111.4	65.8	881.7	11.4	6.9	2.6
2035	100.1	164.9	32.5	68.2	37	1213.3	10.4	6.2	2.2
2303	118.4	222.8	49.2	100.3	9.8	412.9	9.5	4.2	2.8
5551	139.1	213.2	44.4	52.3	45	1538	12.6	6	2.7
7459	124.9	137.6	61	57.6	64.4	540.2	11.4	4.3	2.3
7967	143.3	210.3	36.1	42.9	64	935.7	12.3	5.9	2.4
2439	153.4	207.8	41.3	147.1	227.6	1146.4	9.8	7.7	2.3
5405	100	183.2	44	170.6	40.5	1380.9	13.9	4.8	2.7
5548	108.4	236.3	61.8	139.2	218.8	1652.6	10.4	7.2	2.6
7334	74	-	-	187.2	463.3	1363.7	14.7	3.8	2.7
7399	131.6	138.8	29.9	138.7	153	866.4	9.5	12.4	2
2648	129.6	212.7	22.9	78.2	44.8	1496.7	13.7	7.3	-
2874	107.3	182.9	53	70.6	24.2	1826.6	12.8	4.9	2.4
4297	113.8	236.1	24.3	123.5	29.7	1328.3	14.1	5.8	2.7
5207	179	201.8	23.5	101.2	61.9	758.1	12.2	6.9	2.6
6839	110.2	190	59.6	71.5	87.7	2543.2	9.7	3.9	2.1
7441	137	203.2	21.6	69	40.5	2897.7	12.6	6.5	2.3
9493	172.2	126.5	28.8	41.6	119.3	1715	11.1	8.1	2.4
8301	106.6	169.8	45.1	111.1	29.8	1319.4	11.5	6.6	1.9
9569	130.7	160.6	52	219.1	168.7	3652	11	5.9	2.6
5570	108.3	123.1	56.8	78	77.4	1914	10.5	8	2.2
9407	115.5	164.3	40.5	81	101.8	1601.5	11.7	6.2	2.3
